# N6-Acetyl-L-Lysine and p-Cresol as Key Metabolites in the Pathogenesis of COVID-19 in Obese Patients

**DOI:** 10.3389/fimmu.2022.827603

**Published:** 2022-05-19

**Authors:** Nour Jalaleddine, Mahmood Hachim, Hamza Al-Hroub, Narjes Saheb Sharif-Askari, Abiola Senok, Adel Elmoselhi, Bassam Mahboub, Nimmi Moni Samuel Kurien, Richard K. Kandasamy, Mohammad H. Semreen, Rabih Halwani, Nelson C. Soares, Saba Al Heialy

**Affiliations:** ^1^ College of Medicine, Mohammed Bin Rashid University of Medicine and Health Sciences, Dubai, United Arab Emirates; ^2^ Sharjah Institute for Medical Research, University of Sharjah, Sharjah, United Arab Emirates; ^3^ Department of Medicinal Chemistry, College of Pharmacy, University of Sharjah, Sharjah, United Arab Emirates; ^4^ Department of Pulmonary Medicine and Allergy and Sleep Medicine, Rashid Hospital, Dubai Health Authority, Dubai, United Arab Emirates; ^5^ Centre of Molecular Inflammation Research (CEMIR), and Department of Clinical and Molecular Medicine (IKOM), Norwegian University of Science and Technology, Trondheim, Norway; ^6^ Prince Abdullah Ben Khaled Celiac Disease Research Chair, Department of Pediatrics, Faculty of Medicine, King Saud University, Riyadh, Saudi Arabia; ^7^ Department of Clinical Sciences, College of Medicine, University of Sharjah, Sharjah, United Arab Emirates; ^8^ Meakins-Christie Laboratories, Research Institute of the McGill University Health Center, Montreal, QC, Canada

**Keywords:** SARS-CoV-2, COVID-19, obesity, metabolomics, ultra-high-performance liquid chromatography-mass spectrometry

## Abstract

Despite the growing number of the vaccinated population, COVID-19, caused by the severe acute respiratory syndrome coronavirus 2 (SARS-CoV-2), remains a global health burden. Obesity, a metabolic syndrome affecting one-third of the population, has proven to be a major risk factor for COVID-19 severe complications. Several studies have identified metabolic signatures and disrupted metabolic pathways associated with COVID-19, however there are no reports evaluating the role of obesity in the COVID-19 metabolic regulation. In this study we highlight the involvement of obesity metabolically in affecting SARS-CoV-2 infection and the consequent health complications, mainly cardiovascular disease. We measured one hundred and forty-four (144) metabolites using ultra high-performance liquid chromatography-quadrupole time of flight mass spectrometry (UHPLC-QTOF-MS) to identify metabolic changes in response to SARS-CoV-2 infection, in lean and obese COVID-19 positive (n=82) and COVID-19 negative (n=24) patients. The identified metabolites are found to be mainly correlating with glucose, energy and steroid metabolisms. Further data analysis indicated twelve (12) significantly yet differentially abundant metabolites associated with viral infection and health complications, in COVID-19 obese patients. Two of the detected metabolites, n6-acetyl-l-lysine and p-cresol, are detected only among the COVID-19 cohort, exhibiting significantly higher levels in COVID-19 obese patients when compared to COVID-19 lean patients. These metabolites have important roles in viral entry and could explain the increased susceptibility of obese patients. On the same note, a set of six metabolites associated with antiviral and anti-inflammatory functions displayed significantly lower abundance in COVID-19 obese patients. In conclusion, this report highlights the plasma metabolome of COVID-19 obese patients as a metabolic feature and signature to help improve clinical outcomes. We propose n6-acetyl-l-lysine and p-cresol as potential metabolic markers which warrant further investigations to better understand their involvement in different metabolic pathways in COVID-19.

## Introduction

Infection by the severe acute respiratory syndrome coronavirus 2 (SARS-CoV-2), the virus behind the COVID-19 pandemic, remains a major health concern and global pandemic; despite vaccine roll-out (1–4). Presentations of COVID-19 have ranged from asymptomatic to severe, and sometimes life-threatening. COVID-19 patients may encounter different clinical symptoms (5), including acute respiratory distress syndrome (ARDS), organ malfunctions, or even death in severe cases (6). Obesity, a worldwide health burden contributing to different comorbidities (7–9), has been shown to be a major risk factor for COVID-19 complications and high mortality rate (10–13). Moreover, it has been evidenced that antiviral medication are less effective in obese individuals (14). In spite of the extensive research highlighting obesity as a major risk factor for COVID-19, there remains a need to better understand the mechanisms behind this association. Elucidation of the pathophysiological and molecular mechanisms through which obesity increases the complications related to COVID-19 have the potential to contribute towards improving clinical outcomes in these patients.

Obesity, defined by increased body mass index (BMI) and fat, is a systemic disorder that plays a key role in COVID-19 severity and poor outcome (14–17). Obesity is associated with dysregulated lipid synthesis and clearance, imposing its adverse effects on the immune system, and inducing what is known as chronic meta-inflammation (14–16). Knowing that SARS-CoV-2 manipulates angiotensin converting enzyme 2 (ACE2) receptors for its entry into the host cell, it is evidenced that obese subjects tend to express elevated levels of ACE2, suggesting that ACE2 upregulated expression is due to the dysregulated lipid metabolism pathways (18).Additionally, Al-Benna et al. provided evidence about an increased expression of ACE2 in adipose tissue. Such increase of ACE2 was considered a risk factor for COVID-19 lethality in obese patients (13). Classified as a metabolic syndrome (19), many studies have identified obesity related metabolic signatures as a risk factor of other diseases, including diabetes and cardiovascular disease (20, 21). Of note, viral infections have also shown to induce significant molecular alterations in the host metabolome. Such changes involve various lipid and immune metabolic profiles, including energy mediators. Importantly, it is well established that lipid synthesis and signaling are hijacked by viruses in order to produce lipids for their viral envelopes which allow viral entry, replication, and its release (22). On this basis, using metabolomics as an emerging bioanalytical approach will serve as a useful tool in providing insights into the metabolic changes that occur in COVID-19, and will enable the identification of potential biochemical determinants of obesity involvement in the pathophysiology of COVID-19 (23, 24).

Metabolic profiling of SARS-CoV-2 infection has been previously investigated by several research groups (25–28). A small number of recent studies have highlighted the disruption of different pathways as metabolic features of COVID-19 patients compared to healthy (non-COVID-19) patients (25–28). Dysregulated pathways included lipid metabolism, tryptophan metabolism, nitrogen and fat metabolism, in addition to enhanced oxidative stress and inflammatory biomarkers (26–28). In parallel, it has been shown that SARS-CoV-2 infection and obesity tend to share common pathways associated with glucose and lipid metabolism dysregulation, as determined by *in vitro* and *in silico* approaches. Dysregulated pathways that involved inflammation and immune response, in addition to disrupted cell and tissue homeostasis were identified (18). Despite the identified dysregulated pathways and metabolites in COVID-19, a profound understanding underlying the metabolic regulation of obesity as a major risk to COVID-19 severity remains to be elucidated. Therefore, understanding the metabolomic changes of COVID-19 patients in the context of obesity may provide clues to pathophysiological processes, and better clinical management.

In this study we generated a patient-metabolic profile associated with the body mass index (BMI) *via* untargeted Ultra High-Performance Liquid Chromatography-Quadrupole Time of Flight Mass Spectrometry (UHPLC-QTOF-MS). The detected metabolites tend to be potential biomarkers that may correlate with COVID-19 severity and clinical complications. We highlight the significantly yet differentially altered levels of 12 metabolites out of a total of 144 identified metabolites, in both lean and obese COVID-19 patients. We also evaluated their possible contribution to COVID-19 manifestations in obese patients with a special emphasis on n6-acetyl-l-lysine and p-cresol.

## Materials and Methods

### 1- Participants

A cohort of 82 COVID-19 patients was recruited from the COVID-19 Biobank, through collaboration with University of Sharjah; 2020. Patients were originally recruited from Rashid Hospital, Dubai, UAE. Samples were obtained prior to vaccine development. The studied population was categorized as asymptomatic, mild, moderate and severe. Symptoms included fever, cough, nausea, vomiting, dyspnea, diarrhea, and confusion. These patients were further stratified according to their body mass index (BMI); obese (n=28), overweight (n=19) and lean (n=35) COVID-19 patients. Patients with BMI ≥ 30 kg/m^2^, BMI = 25-29.9 kg/m^2^, and BMI < 25 kg/m^2^ are classified as obese, overweight, and lean (non-obese), respectively. Additionally, a cohort of 24 non-COVID-19 patients (controls) were recruited from the Biobank. Control patients were also stratified according to BMI; obese (n=15), overweight (n=4) and lean (n=5) non-COVID-19 patients. **Table 1** describes the patients’ characteristics enrolled in this study. A further classification was employed highlighting the notion of COVID-19 patients with increased BMI tend to share more of moderate and severe symptoms, as represented in the severe and moderate categories; **Table 2**.

**Table 1:** Summary of subjects characteristics enrolled in the study.

Subjects Characteristics		
	Non-COVID-19	COVID-19
***Subjects (N)***	24	82
***Age (years)***	48.2 ± 13.6	47 ± 15.86
***Gender, M/F***	12/12	64/18
***BMI***	30.31 ± 3.7	26.1 ± 5.05

BMI, Body Mass Index; M, Male; F, Female; N, Number of Subjects; ± indicates Standard Deviation.

**Table 2:** Severity and BMI categories of COVID-19 patients.

COVID-19 patients				
Severity	N, (%)	Obese N, (%) (BMI≥ 30 kg/m^2^)	Overweight N, (%) (BMI = 25-29kg/m^2^)	Lean N, (%) (BMI< 25 kg/m^2^)
**Mild**	26, (31%)	8, (31%)	8, (31%)	10 (38%)
**Moderate**	11, (13%)	5, (46%)	2, (18%)	4, (36%)
**Severe**	17, (20%)	7, (42%)	5, (29%)	5, (29%)
**Asymptomatic**	28, (34%)	8, (29%)	4, (14%)	16, (57%)
**Total N, (%)**	82, (100%)	28, (34%)	19, (23%)	35, (43%)

BMI, Body Mass Index; N, Number of Subjects.

Selected participants gave written informed consent before recruitment. The study was approved by the ethics committee (DSREC-04/2020_19) of the Dubai Scientific Research Ethics (Dubai Health Authority; DHA) in the United Arab Emirates.

### 2- Sample Collection and Preparation

Fasting blood samples were collected from subjects for clinical profiling. The same blood samples were used later for plasma extraction and further analysis. Briefly, 8 ml of peripheral venous blood were collected in Ethylenediamine Tetra Acetic Acid (EDTA)-blood collection tubes and were processed within 24 hours. For plasma collection, blood was diluted with (1x) Phosphate Buffered Saline (Cat. #: RNBH9883, Sigma-Aldrich, USA) containing 2% human fetal bovine serum (FBS; Cat. #: F9665, Sigma-Aldrich, USA), in a 1:1 ratio, mixed thoroughly, and layered onto a HISTOPAQUE^®^-1077gradient media (Cat. #: RNBK2836, Sigma-Aldrich, USA). To separate plasma from other blood components, centrifugation was performed at 400 × *g* for 30 minutes; room temperature. Plasma was then collected and stored at − 80°C until further use.

### 3- Untargeted UHPLC-QTOF-MS Analysis and Metabolite Identification

Samples were analyzed using Ultra High-Performance Liquid Chromatography [UHPLC; (UHPLC; ELUTE autosample, from Bruker (Bremen-Germany)] coupled to Trapped Ion Mobility (timsTOF) Mass spectrometer [Bruker Daltonics GmbH & Co. KG, (Bremen, Germany)]. Briefly, 150 µl of each sample was aliquoted and suspended in 450 µl of methanol. After vortex for 1 minute, the samples were incubated at -20°C for 2 hours. Samples were then centrifuged at 14,000 rpm for 15 minutes. The supernatant was transferred into new Eppendorf tubes for vacuum freeze-drying speed vacuum evaporation at 35 – 40°C. A quality control (QC) samples were prepared by pooling the same volume of each sample to evaluate the reproducibility of the analysis. Extract samples were reconstituted by 250 ul of 0.1% formic acid in Deionized Water-LC-MS CHROMASOLV (Honeywell) and loaded onto the Autosampler (ELUTE UHPLC). Metabolites were analyzed in auto MS/MS positive scan mode within the range of 20-1300 m/z utilizing electrospray ionization using Bruker Compass HyStar 5.0 SR1 Patch1 (5.0.37.1), Compass 3.1 for otofSeries, otofControl Version 6.0. Metabolites were potentially identified according to the endogenous MS database by accurate masses. At the same time, the metabolites that matched with the spectra in the fragment database were confirmed at the tandem mass spectrometry level.

For analysis purposes, we have managed to combine the detected metabolite intensity from overweight and obese subjects since there was a significant linear correlation between the development of COVID-19 severe complications and hospitalization in subjects with BMI ≥ 23 kg/m^2^ (29–32).

### 4- Enrichment Analysis

To gain insight into the metabolomic mechanisms, pathway analysis and metabolite set enrichment analysis (MESA) were implemented using MetaboAnalyst-5.0, a web-based metabolomics data analysis software (33). Over Representation Analysis (ORA) was performed using the hypergeometric test to evaluate whether a metabolite set is represented more than expected by chance within the given compound list. The concentrations of statistically significant metabolites that have *p <*0.05 in *t*-test were used for quantitative pathway enrichment analysis.

### 5- Statistical Analysis

Microsoft Excel and GraphPad Prism software were used to perform statistical analysis. Results are expressed as individual data or as median ± standard deviation. Data series were compared by calculating the relative variability of data with respect to the mean *via* Coefficient of variation (CV). A CV>20 was not included in further analysis. Prior to analysis, data were tested for normality using SharpiroWilk’s and D’Agostino and Pearson omnibus normality tests. Data were analyzed using either Mann-whitney t-test or ordinary one-way ANOVA. Statistical significance was determined by Mann-Whitney *t*-test followed by Tukey Post -Hoc or Welch’s correction tests. Differences among groups was assessed by one-way analysis of variance (ANOVA with Tukey’s *post hoc* test or Dunn’s multiple comparison test and Kruskal-Walis test), with 95% probability level; *p <*0.05 was considered significant.

## Results

### Profound Metabolomics Alterations Associated With COVID-19 Lean and Overweight-Obese Patients

**Figure 1:**
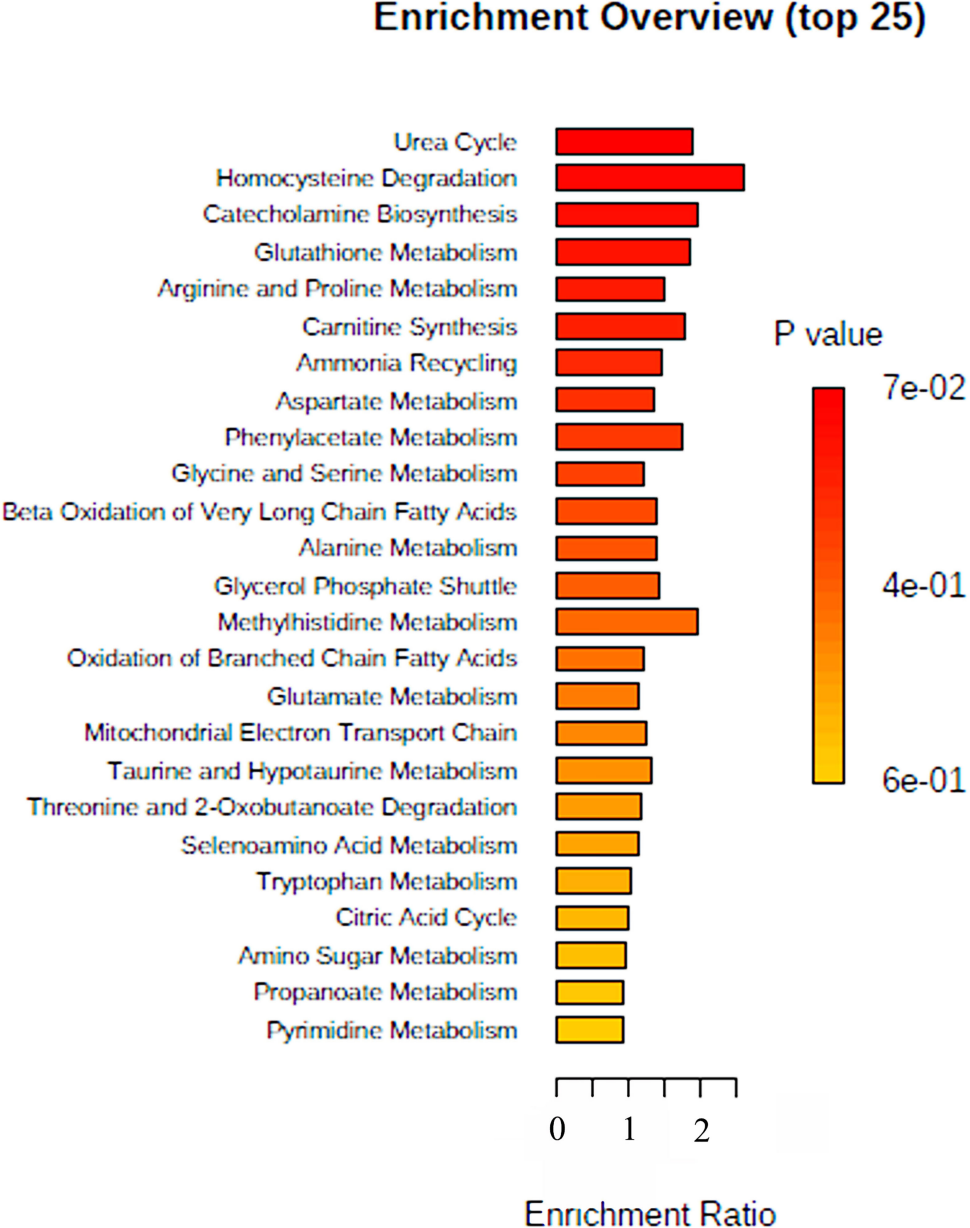
Summary of Metabolomic Set Enrichment Analysis Plot. Metabolomic Set Enrichment Analysis (MSEA) showing the most altered functional metabolic pathways in COVID-19 patients. The graph was obtained using the online tool MSEA by plotting on the y-axis the -log of *p* values from pathway enrichment analysis.

This study aimed to investigate the plasma metabolome profiling of COVID-19 patients using UHPLC–MS/MS, to reveal the significantly altered metabolites among non-COVID-19 and COVID-19 in lean and overweight-obese patients. Our profiling detected 144 metabolites in COVID-19 patients and 147 metabolites in non-COVID-19 patients; listed in **Supplementary Table 1** (**Table S1**). To gain insight into the metabolic mechanisms by which the 144 metabolites are involved, data extracted from the MetaboAnalyst indicated that these metabolites had a major impact in different pathways (**Figure 1**), when comparing COVID-19 patients to non-COVID-19 patients. The top or highly identified pathways were mainly associated with glucose, energy and steroid metabolisms. These include homocysteine degradation, methyl histidine metabolism, and catecholamine biosynthesis, in addition to other enriched pathways such as urea cycle and glutathione and arginine and proline metabolisms (**Figure 1**).

**Figure 2:**
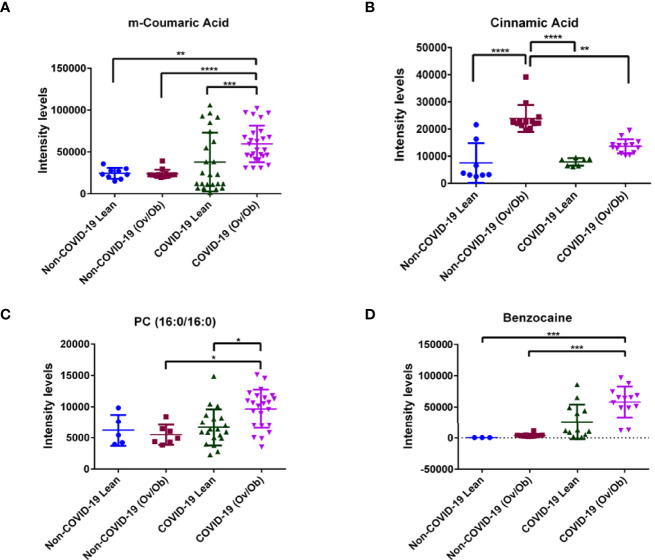
Alteration in plasma metabolite levels of COVID-19 lean and obese patients compared to non-COVID-19 lean and obese patients. Plasma levels of different metabolites **(A)** m-Coumaric acid **(B)** Cinnamic acid **(C)** Phosphatidylcholine [PC (16:0/16:0)] and **(D)** Benzocaine, were significantly different in COVID-19 lean and overweight-obese patients when compared to controls (non-COVID-19). Analysis was performed using one-way ANOVA with Dunn’s multiple comparison and Kruskal-Walis tests; *P < 0.05; **P < 0.01; ***P < 0.001; ****P < 0.0001. The *y* axis in the dot plots indicates arbitrary units of median intensity.

Next, we analyzed the levels of the detected metabolites among COVID-19 lean and overweight and obese subjects compared to non-COVID-19 lean, overweight and obese subjects. Our results showed that among the detected 144 metabolites in COVID-19 patients, the levels of 3 metabolites having antioxidant and anti-inflammatory properties (m-coumaric, Phosphatidylcholine (PC (16:0/16:0)), and benzocaine) were significantly upregulated in COVID-19 lean and COVID-19 overweight-obese patients when compared to non-COVID-19 counterparts (**Figure 2**). The phenolic compound m-coumaric acid showed significantly upregulated levels in COVID-19 overweight-obese (*p* < 0.01; *p* < 0.0001 respectively) as compared to non-COVID-19 subjects. Also, it displayed significantly higher levels in COVID-19 obese patients (*p* < 0.001) as compared to COVID-19 lean patients (**Figure 2A**). Additionally, our data also revealed another phenolic compound, cinnamic acid, having significantly higher levels (*p* < 0.0001) in both non-COVID-19 and COVID-19 overweight-obese patients when compared to their lean counterparts. On the other hand, cinnamic acid displayed significantly lower levels (*p* < 0.01) in COVID-19 obese individuals than in non-COVID-19 obese individuals (**Figure 2B**). Our data also showed significantly higher levels of the Phosphatidylcholine glycerophospholipid [PC (16:0/16:0)] in COVID-19 overweight-obese patients as compared to COVID-19 lean and non-COVID-19 overweight-obese (*p* < 0.05; **Figure 2C**). Although benzocaine was identified by our human metabolite data base (HMDB), it’s not a cell metabolite and rather classified as local anesthetic. Benzocaine showed significantly higher levels (*p* < 0.001) in COVID-19 overweight-obese patients as compared to non-COVID-19 lean and overweight-obese subjects (**Figure 2D**). Overall, these data indicate that both SARS-CoV-2 infection and obesity tend to significantly affect the host metabolome.

### Metabolic Signatures Associated With Viral Infection and Disease Complications in COVID-19 Overweight-Obese Compared to COVID-19 Lean Patients: N6-Acetyl-L-Lysine and p-Cresol as Key Mediators

**Figure 3:**
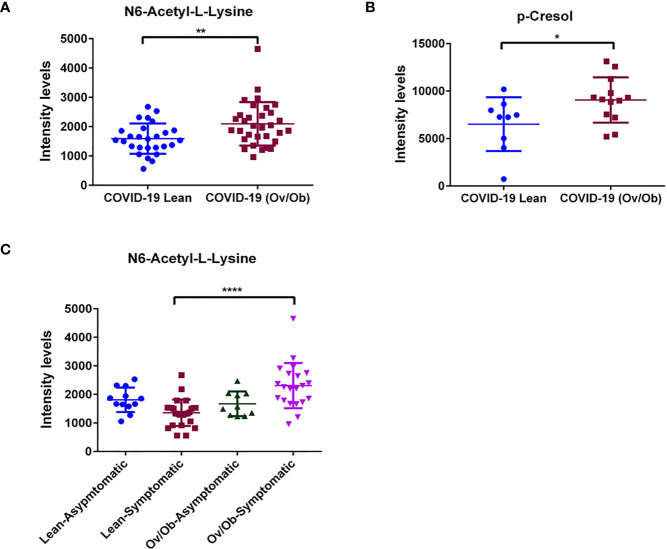
Upregulated levels of plasma metabolites as markers of viral function and adverse complications in COVID-19 obese patients. Plasma levels of **(A)** N6-Acetyl-L-Lysine were significantly upregulated when comparing COVID-19 obese and overweight patients to COVID-19 lean patients. Similarly, significant increase in plasma levels of **(B)** p-Cresol in COVID-19 overweight-obese patients. Analysis was performed using Mann Whitney *t*-test with or without Welch’s correction test. **(C)** Plasma metabolite levels of N6-acetyl-l-lysine displayed altered levels that correlated with the occurrence of symptoms in COVID-19 overweight-obese patients. Analysis was performed using one-way ANOVA followed by Dunn’s multiple comparison and Kruskal-Wallis test*post hoc*; *P < 0.05; **P < 0.01; ****P < 0.0001.

**Figure 4:**
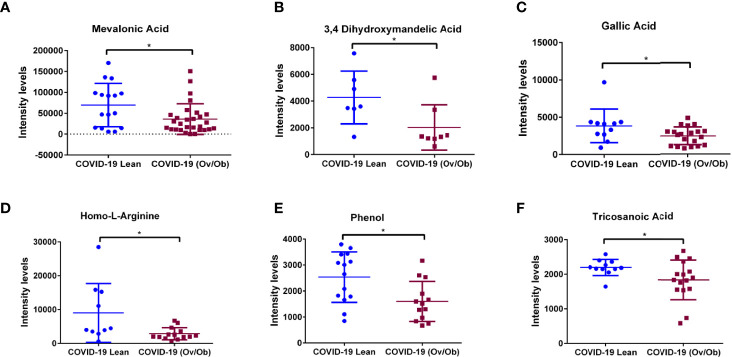
Downregulation of plasma metabolites as markers of COVID-19 health complications in obese patients. Plasma levels of **(A)** Mevalonic acid, **(B)** 3,4-Dihydroxymandelic acid, **(C)** Gallic acid, **(D)** Homo-L-Arginine, **(E)** Phenol, and **(F)** Tricosanoic acid displayed significantly lower levels in COVID-19 overweight-obese patients compared to COVID-19 lean patients. Analysis was performed using Mann Whitney *t*-test with or without Welch’s correction test; *P < 0.05.

Our analysis identified a set of metabolites that were significantly altered in COVID-19 overweight-obese patients when compared to COVID-19 lean patients. Data showed significantly upregulated levels of n6-acetyl-l-lysine (*p* < 0.01; **Figure 3A**), a metabolite that belongs to a class of organic compounds known as l-alpha-amino acids (34), and is known for its role in chronic inflammation and viral function induction (34, 35). In addition to n6-acetyl-l-lysine, data showed significantly upregulated levels of the protein-bound uremic retention solute p-cresol (*p* < 0.05) in COVID-19 overweight-obese patients when compared to COVID-19 lean patients (**Figure 3B**). P-cresol is known to associate with increased admissions of hospitalizations due to infections (36). Moreover, its high levels is usually correlated with chronic inflammation and cardiovascular diseases (37). Interestingly, n6-acetyl-l-lysine and p-cresol metabolites are not among the detected metabolites in non-COVID-19 patients; they were only detected in COVID-19 patients. On the other hand, data showed significantly lower levels of mevalonic acid, phenol, 3,4- dihydroxymandelic acid, gallic acid, homo-l-arginine (*p* < 0.05; **Figures 4A–F**) in COVID-19 overweight-obese compared to COVID-19 lean patients. Interestingly, these metabolites tend to share anti-inflammatory and anti-oxidant properties (38).

### Profound Metabolomics Alterations Associated With COVID-19 Symptomatic Manifestation in COVID-19 Lean and COVID-19 Overweight-Obese Patients

We assessed the involvement of the 12 significantly altered metabolites in the symptomatic phenotype presented by COVID-19 patients. Interestingly, n6-acetyl-l-lysine showed to be also significantly involved in the symptoms of COVID-19 patients. Our analysis showed a significantly upregulated level of n6-acetyl-l-lysine (*p* < 0.0001) in symptomatic COVID-19 overweight-obese patients compared to symptomatic COVID-19 lean patients. (**Figure 3C**). This potentially signifies the involvement of n6-acetyl-l-lysine in obesity-induced symptoms in COVID-19 patients. No significant involvement was detected regarding the rest of the metabolites.

## Discussion

The findings from this study unravel the effect of obesity on the metabolome profiling of COVID-19 patients. We have identified a total of 144 metabolites in COVID-19 patients from which 12 metabolites displayed significantly altered levels between lean and overweight-obese COVID-19 patients. We also highlight that n6-acetyl-l-lysine and p-cresol are metabolic signatures of COVID-19 and were significantly displaying higher levels in overweight-obese COVID-19 patients compared to lean COVID-19 patients. Despite that these COVID-19 patients present different or no other metabolic complications, yet our findings suggest that n6-acetyl-l-lysine and p-cresol are rather influenced by COVID-19. The detected metabolic signature of these patients may shed light on the involvement of obesity in the progression and severity of COVID-19 disease.

In this study we showed plasma metabolic profiling from lean and overweight-obese COVID-19 patients. Upregulated levels of n6-acetyl-l-lysine and p-cresol were evidenced in overweight-obese COVID-19 patients. Lysine acetylation is a novel post-translational pathway that is mainly induced by obesity, and has been shown to regulate enzyme activities involved in fatty acid and glucose metabolisms (39). This mechanism involves the transfer of an acetyl group from acetyl Coenzyme A (acetyl-CoA), key mediator of protein acetylation and a regulator of metabolism, targeting lysine amino group (40, 41). Interestingly, acetylation of several proteins has been evidenced at virus interacting domains and has been shown to affect viral replication, thus indicating a crucial role in viral infection. It was observed in Influenza virus and Zaire Ebolavirus that the nucleocapsids (N) were acetylated, suggesting that these modifications contribute to the molecular events involved in viral replication. Similarly, SARS-CoV and SARS-CoV-2 N proteins are shown to be strongly acetylated by human histone acetyltransferases, forming acetyl-lysine residues. These acetylated lysine residues are mainly localized at the N- terminal and C-terminal functional interacting sites of RNA and the membrane (M)-protein (35), which was shown to play a role in viral infectivity and antigenicity (42). Although the bi-directional virus-host and chromatin regulation are not yet fully understood, histone modifications have been proven to affect viral chromatin which possibly influence epigenetic factors that mediate viral survival and function, allowing it to escape the immune system (43). Generally, the acetylation of these proteins is crucial for viral pathogenesis (35). Additionally, findings from *in vitro* work with human lung adenocarcinoma epithelial cells (Calu-3 cells) also indicates that uremic toxin p-cresol contributes to the induction of cytokine storm in response to SARS-CoV. Culturing Calu-3 cells with p-cresol induced responses that exhibited enhanced expression and secretion of the proinflammatory tumor necrosis factor (TNF-α), Interleukins (IL-6 and IL-1β), and interferon gamma (IFNγ) cytokines (36). This suggests that our identified metabolic signatures, N6-acetyl L-Lysine and p-Cresol, are potentially involved in inducing cytokine storm. These inflammatory cytokines are suggested to be markers of acute systemic inflammatory syndrome that can be measured in COVID-19 patients with increased severity (44, 45). p-Cresol has been evidenced to inhibit macrophage function and leukocyte migration involved in cytokine-stimulated cells (46). Moreover, p-cresol has shown to significantly upregulate ACE2 and TMPRSS2, the receptors for SARS-CoV-2, which reflects more viral entry into the host cell (36). Overall, this suggests that n6-acetyl-l-lysine and p-cresol are two key metabolites that may play crucial roles in the COVID-19 pathogenesis, where obesity could be a triggering factor. In addition to the upregulated levels of n6-acetyl-l-lysine and p-cresol, our data also displayed significantly lower levels of phenol, gallic acid, 3,4-dihydroxymandelic acid, mevalonic acid and homo-l-arginine in overweight-obese COVID-19 patients, which tend to have antiviral properties (47–50). Interestingly, among these metabolites is the mevalonic acid, which indicates the participation of the mevalonate pathway in our study. It is known that cholesterol synthesis is part of the acetyl-CoA-mevalonate pathway, keeping in mind that the adipose tissue is a major site for cholesterol storage. Disruption or inhibition of this pathway is mainly correlated with glucose and lipid metabolic disorders, in addition to hepatomegaly (51, 52). Intriguingly, the lipid metabolism plays a crucial role in the viral life cycle as a host machinery for viral replication, impairing the host immune response. SARS-CoV-2 has been evidenced to hijack the mevalonate pathway, leading to activation of the inflammasome *via* cytokine release. This indicates that this pathway is involved in the pathogenesis of COVID-19; as determined by *in silico* assays (22, 53). Overall, we highlight the involvement of obesity in COVID-19 disease *via* enhancing viral propagation due to the altered levels of the host metabolome which may warrant further investigation.

Besides their possible effect on viral load, the significantly altered levels of the detected metabolites might be also mediating the health complications observed in COVID-19 overweight-obese patients, which then leads to poor prognosis. The increased plasma levels of n6-acetyl-l-lysine and p-cresol metabolites are mainly attributed to heart and kidney failure (34), complications that have been observed in COVID-19 patients (54). Protein acetylation are shown to regulate multiple signaling pathways of inflammation as well as metabolic enzymes involved in glycolysis, tricarboxylic acid and urea cycles and fatty acid and glycogen metabolism (55), linking them to metabolic dysfunction and diseases. Interestingly, lysine acetylation has been linked to the regulation of cardiac energy metabolism and insulin signalling. In the case of obesity, the elevated levels of free fatty acids uptake and β oxidation, provides an abundance of the acetyl-CoA, leading to mitochondrial dysfunction; a unique feature of obesity and/or diabetes (39). Of note, chronically increased fatty acid uptake and β- oxidation accompanied with insulin resistance enhances protein lysine acetylation, which in turn induces mitochondrial protein hyperacetylation, leading to cardiac malfunction or disease (56, 57). Similarly, p-cresol, a protein-bound uremic retention solute mainly produced in the human intestine due to bacterial fermentation (58), is a novel cardiovascular risk factor that contributes to atherosclerosis and thrombosis in uremic patients. This is partially due to the production of reactive oxygen species (ROS) and inflammation, in addition to endothelial and vascular dysfunction associated with atherosclerosis-related enzymes (37, 59). In parallel, our data revealed the significant decrease in homo-l-arginine, mevalonic acid, phenol, 3,4- dihydroxymandelic acid, and gallic acid plasma levels in overweight-obese COVID-19 patients. These metabolites, whether at the plasma level or as a pretreatment, are known to exhibit anti-inflammatory and antioxidant properties (38, 39). Among these, homo-L-arginine, a naturally occurring, non-proteinogenic, cationic amino acid catalyzed by the liver *via* glycine amidinotransferase (AGAT); an enzyme from the creatine biosynthesis pathway (60) is known to exert protective roles on the endothelial function and energy metabolic processes in different organs. However, its low levels have been attributed to diabetes, kidney diseases, and to cardiovascular diseases with high mortality rates (60–62). In addition, homo-l-arginine have shown to have protective functions against progression of pulmonary fibrosis. This makes it an interesting metabolite to monitor for progression to pulmonary fibrosis. Generally, arginine and its derivatives are part of the urea cycle metabolic pathway, by which its dysfunction is known to correlate with lung disease (63). This could also explain our MSEA analysis, which displayed the urea cycle to be one of the top enriched pathways observed in our study. Interestingly, our analysis displayed homocysteine degradation pathway to be the most enriched pathway, highlighting its involvement as a potential predictor of cardiovascular risk in COVID-19 patients (64). Overall, our data explains how these metabolites are involved complications seen in overweight-obese COVID-19 patients.

Our assessment also displayed plasma metabolite profiling from overweight-obese and lean COVID-19 patients of differentially altered levels of m-coumaric, cinnamic, phosphatidylcholine [PC (16:0/16:0)] and benzocaine when compared to non-COVID-19 lean and overweight-obese patients. The phenolic compounds, m-coumaric acid and cinnamic acid exhibit anti-inflammatory and antioxidant properties, in addition to their immunomodulatory functions (65–67). These compounds were shown to serve as a therapy for obesity-related health complications. As natural compounds, these hydroxycinnamic derivatives were shown to protect against obesity associated health complications by targeting the proinflammatory tumor necrosis factor (TNF-α) and nuclear factor κB (NF-κB) pathways *via* reducing their expression (68). Despite their protective properties, our data revealed higher plasma levels of these compounds in overweight-obese COVID-19 patients as compared to lean- COVID-19 patients. It is clear that SARS-CoV-2 like many other viruses imposes its molecular alterations in the host metabolome (22) and a possible functional compensation might be taking place. Additionally, our data displayed higher levels of the glycerophospholipid PC (16:0/16:0) and benzocaine in overweight-obese COVID-19 patients. These metabolites are shown to participate in cardiovascular and neurodegenerative diseases by inducing inflammation (69, 70), and in cancer by enhancing the severity of malignancy (71). In a case study, benzocaine was shown to induce methemoglobinemia after its administration as premedication for transesophageal echocardiography (72).

**Figure 5:**
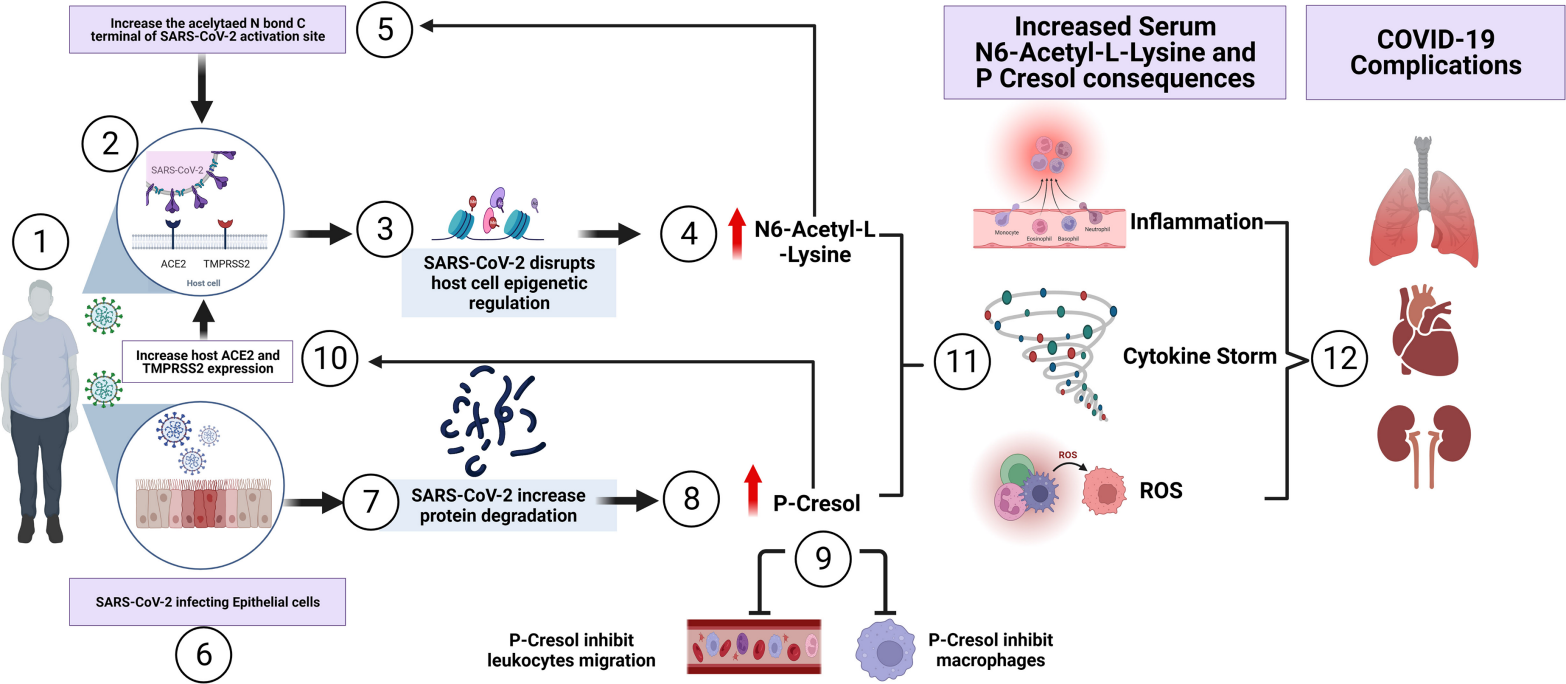
Summary of the proposed involvement of N6-Acetyl-L-Lysine and p-Cresol as key metabolites of COVID-19 pathogenesis observed in COVID-19 overweight-obese patients. (1) SARS-CoV-2 access the airway of an overweight-obese patient by binding to the host cell. (2) The virus utilizes ACE2 receptor for its entry and the serine protease TMPRSS2 for S protein priming, contributing to rapid spread of the virus in infected patients. (3) Inside the host cell, SARs-CoV-2 influence epigenetic factors that manipulates viral survival and replication, leading to metabolic dysregulation. (4) The increase of N6-Acetyl-L-Lysine, as a key metabolite of COVID-19 pathogenesis in overweight-obese patients, induces molecular alterations in SARS-CoV-2 Nucleoplasmid and RNA at functional interacting sites. This in turn creates a positive feedback loop through the induction of viral replication and infectivity. In another mechanism, (5) and (6) SARS-CoV-2 invasion of epithelial cells, leads to the (7) degradation of proteins and thus accumulating as a by-product toxin. Of these, is the uremic toxin (8) P-Cresol, another key metabolite involved in the pathogenesis of COVID-19 disease, displayed increased intensity in overweight-obese COVID-19 patients. Elevated p-Cresol (9) is shown to inhibit macrophage activity and leukocyte cell adhesion, which leads to decrease of immune response, as seen in COVID-19 patients. (10) In another mechanism, p-Cresol increases the expression of ACE2 receptor and TMPRSS2, thus enhancing viral entry and load. (11) The increase of N6-Acetyl-L-Lysine and p-Cresol resulted in the induction of inflammation, cytokine storm and reactive oxygen species (ROS) production. This subsequently led to the development of (12) severe health complications which involves pulmonary fibrosis, cardiovascular diseases and kidney failure, and possibly admission to intensive care unit (ICU).

In summary, our investigation, to our knowledge, is the first study to characterize the plasma metabolome of COVID-19 overweight-obese patients. Such metabolic signature could possibly be used as a clinical indicator of health complications observed in obese COVID-19 patients. We propose the correlation of two metabolites, n6-acetyl-l-lysine and p-cresol, in having crucial roles in COVID-19 poor prognosis of obese individuals, as demonstrated in **Figure 5**. Despite the limited access to non-vaccinated COVID-19 patient samples, in the present study we provide a proof-of-principle evidence for the feasibility of understanding obesity induced metabolic dysregulation in the pathogenesis of COVID-19, without vaccination being a co-founding factor. Future investigations are required to gain more mechanistic insights with regards to the actual involvement of such metabolites in pathways driving the occurrence of complications in obese COVID-19 individuals. Also, future directions should be addressing the metabolome of vaccinated COVID-19 patients, considering the involvement of obesity as a risk factor to adverse complications in COVID-19 disease.

## Data Availability Statement

The original contributions presented in the study are included in the article/**Supplementary Material**. Further inquiries can be directed to the corresponding authors.

## Ethics Statement

This study involved human patients (non-COVID-19 and COVID-19). Participants gave written informed consent before recruitment. The study was approved by the ethics committee (DSREC-04/2020_19) of the Dubai Scientific Research Ethics (Dubai Health Authority; DHA) in the United Arab Emirates. The patients/participants provided their written informed consent to participate in this study.

## Author Contributions

Conceptualization, SA. Experimental design, NJ, SA, MH, HA-H. NS. Sample collection, BM, NS, RH and NS-A. Formal analysis, NJ. Supervision of analysis, RKK. Resources, SA. Data curation, NJ and SA. Writing—original draft preparation, NJ. Writing—review and editing, NJ, SA, NS, MS, AS, RH, RK, MH, NS-A, BM, NS, and HA-H. Supervision, SA. Funding Acquisition: Dubai Healthcare Authority (DHA). All authors read and approved the final manuscript

## Funding

Funding was provided by the Mohammed Bin Rashid University of Medicine and Health Sciences Internal Research Grant (MBRU-CM-RG2020-06) and by COVID-19 research grant (COV19-0307), University of Sharjah, UAE and Al Jalila Foundation Seed Grant (AJF202019) to RH; and by Prince Abdullah Ben Khalid Celiac Disease Research Chair, under the Vice Deanship of Research Chairs, King Saud University, Riyadh, Kingdom of Saudi Arabia.

## Conflict of Interest

The authors declare that the research was conducted in the absence of any commercial or financial relationships that could be construed as a potential conflict of interest.

## Publisher’s Note

All claims expressed in this article are solely those of the authors and do not necessarily represent those of their affiliated organizations, or those of the publisher, the editors and the reviewers. Any product that may be evaluated in this article, or claim that may be made by its manufacturer, is not guaranteed or endorsed by the publisher.
