# Supplementary material for: N6-Acetyl-L-Lysine and p-Cresol as Key Metabolites in the Pathogenesis of COVID-19 in Obese Patients
Source: Front Immunol. 2022 May 19;13:827603. doi: 10.3389/fimmu.2022.827603 (PMC9161728; doi:10.3389/fimmu.2022.827603)
Supplement: Supplementary file 1 [file Table_1.docx]

**Table S1: Metabolic Profile of non-COVID-19 and COVID-19 patients.**

|  | **Non-COVID-19** | **COVID-19** |
| --- | --- | --- |
| 1- | 1-Methyladenosine | 1-Methylhistidine |
| 2- | 1-Methylhistidine | 11a-Hydroxyprogesterone |
| 3- | 2,3-Diaminopropionic acid | 2,5-Furandicarboxylic acid |
| 4- | 2,5-Furandicarboxylic acid | 2-Aminobenzoic acid |
| 5- | 2-Hydroxycaproic acid | 2-Ketobutyric acid |
| 6- | 2-Pyrrolidinone | 2-Pyrrolidinone |
| 7- | 3,4,5-Trimethoxycinnamic acid | 3,4,5-Trimethoxycinnamic acid |
| 8- | 3,5-Dimethoxyphenol | 3,4-Dihydroxymandelic acid |
| 9- | 3-Hexenedioic acid | 3,4-Dihydroxyphenylglycol |
| 10- | 3-Hydroxyanthranilic acid | 3,5-Dimethoxyphenol |
| 11- | 3-Indolepropionic acid | 3-Hexenedioic acid |
| 12- | 3-Methylindole | 3-Indolepropionic acid |
| 13- | 3-Methylxanthine | 3-Methoxyphenylacetic acid |
| 14- | 4-Aminophenol | 3-Methylindole |
| 15- | 5-Hydroxy-L-tryptophan | 3-Pyridylacetic acid |
| 16- | 5-Hydroxyindoleacetic acid | 4,5-Dihydroorotic acid |
| 17- | 5-Methoxytryptophol | 4-Aminophenol |
| 18- | 7-Methylguanine | 5-Aminolevulinic acid |
| 19- | 9-Methyluric acid | 5-Dodecenoic acid |
| 20- | Acetaminophen | 5-Hydroxyindoleacetic acid |
| 21- | Acetaminophen glucuronide | 7,8-Dihydrobiopterin |
| 22- | Acetic acid | Acetaminophen |
| 23- | Acetone | Acetic acid |
| 24- | Adenine | Adenine |
| 25- | Adenosine monophosphate | Adenosine monophosphate |
| 26- | Allantoin | ADP |
| 27- | Alpha-ketoisovaleric acid | Alloxan |
| 28- | Alpha-N-phenylacetyl-L-glutamine | Alpha-hydroxyhippuric acid |
| 29- | Aniline | Alpha-N-phenylacetyl-L-glutamine |
| 30- | Aspartame | Aspartame |
| 31- | Azythromycin | Asymmetric dimethylarginine |
| 32- | Benzaldehyde | Benzocaine |
| 33- | Benzocaine | Benzoic acid |
| 34- | Benzoic acid | Caffeine |
| 35- | Cadaverine | Cinnamic acid |
| 36- | Caffeine | Cis-Aconitic acid |
| 37- | Cinnamic acid | Citramalic acid |
| 38- | Cis-Aconitic acid | Citric acid |
| 39- | Cortisol | Citrulline |
| 40- | Creatine | Cortisol |
| 41- | Creatinine | Creatine |
| 42- | Cytosine | Creatinine |
| 43- | D-Alanine | Cytosine |
| 44- | D-Cysteine | D-Alanine |
| 45- | D-Serine | D-Arabitol |
| 46- | Deoxycholic acid glycine conjugate | D-Glucurono-6,3-lactone |
| 47- | Deoxyguanosine | Dehydroascorbic acid |
| 48- | Diacetyl | Deoxycholic acid glycine conjugate |
| 49- | DL-2-aminooctanoic acid | Dihydrothymine |
| 50- | Elaidic acid | DL-2-aminooctanoic acid |
| 51- | Gluconic acid | DL-a-glycerophosphate |
| 52- | Glucosamine | DUMP |
| 53- | Glycocholic acid | Elaidic acid |
| 54- | Glycyl-L-leucine | Epinephrine |
| 55- | Glycylproline | Epitestosterone |
| 56- | Guanine | Gallic acid |
| 57- | Guanosine | Gluconolactone |
| 58- | Guanosine monophosphate | Glucosamine |
| 59- | Hippuric acid | Glutathione |
| 60- | Homoveratric acid | Glycerophosphocholine |
| 61- | Hypoxanthine | Glycine |
| 62- | Indole | Glycocholic acid |
| 63- | Indole-3-carbinol | Glycyl-L-leucine |
| 64- | Indoleacetic acid | Glycylproline |
| 65- | Indoleacrylic acid | Guanidine |
| 66- | Inosine | Heptadecanoic acid |
| 67- | Iodotyrosine | Hippuric acid |
| 68- | Isobutyric acid | Homo-L-arginine |
| 69- | Isovalerylcarnitine | Homocysteine |
| 70- | Kynurenic acid | Homoveratric acid |
| 71- | L-Acetylcarnitine | Hypoxanthine |
| 72- | L-Arabinose | Imidazole |
| 73- | L-Aspartyl-L-phenylalanine | Indole |
| 74- | L-Carnitine | Indole-3-carbinol |
| 75- | L-Cystine | Indoleacetic acid |
| 76- | L-Glutamine | Indoleacrylic acid |
| 77- | L-Histidine | Indolelactic acid |
| 78- | L-Kynurenine | Indoxyl sulfate |
| 79- | L-Lysine | Inosinic acid |
| 80- | L-Malic acid | Isobutyric acid |
| 81- | L-Methionine | Isovalerylcarnitine |
| 82- | L-Norleucine | Kynurenic acid |
| 83- | L-Phenylalanine | L-Acetylcarnitine |
| 84- | L-Proline | L-Arginine |
| 85- | L-Tryptophan | L-Aspartyl-L-phenylalanine |
| 86- | L-Valine | L-Carnitine |
| 87- | Levofloxacin | L-Glutamine |
| 88- | Linoleic acid | L-Histidine |
| 89- | m-Coumaric acid | L-Kynurenine |
| 90- | Maltotetraose | L-Lysine |
| 91- | N,N-Dimethylformamide | L-Methionine |
| 92- | N-Acetyl-L-alanine | L-Norleucine |
| 93- | N-Acetylneuraminic acid | L-Phenylalanine |
| 94- | N-Acetylputrescine | L-Proline |
| 95- | N-Acetylserotonin | L-Sorbose |
| 96- | N-Methylhydantoin | L-Tryptophan |
| 97- | N1-Acetylspermine | L-Tryptophan |
| 98- | Niacinamide | L-Valine |
| 99- | Nicotinuric acid | Linoleic acid |
| 100- | Normetanephrine | m-Coumaric acid |
| 101- | Nutriacholic acid | Maltitol |
| 102- | o-Tyrosine | Mevalonic acid |
| 103- | Oleanolic acid | N-Acetylserotonin |
| 104- | Oxalacetic acid | N-Methylhydantoin |
| 105- | Oxypurinol | N1-Acetylspermine |
| 106- | p-Aminobenzoic acid | N6-Acetyl-L-lysine |
| 107- | Pantothenic acid | Niacinamide |
| 108- | Paracetamol sulfate | Nicotinuric acid |
| 109- | Paraxanthine | o-Tyrosine |
| 110- | PC (16:0/16:0) | Orotic acid |
| 111- | Phenylacetic acid | p-Cresol |
| 112- | Phenylpropiolic acid | Pantothenic acid |
| 113- | Phosphoric acid | Paraxanthine |
| 114- | Phosphorylcholine | PC (16:0/16:0) |
| 115- | Picolinic acid | Phenol |
| 116- | Pipecolic acid | Phenylacetaldehyde |
| 117- | Propanal | Phenylpropiolic acid |
| 118- | Protocatechuic acid | Phosphoric acid |
| 119- | Pyridine | Pipecolic acid |
| 120- | Pyridoxal 5'-phosphate | Propanal |
| 121- | Pyridoxamine | Protocatechuic acid |
| 122- | Pyrocatechol | Pyridine |
| 123- | Pyroglutamic acid | Pyridoxal 5'-phosphate |
| 124- | Quinaldic acid | Pyroglutamic acid |
| 125- | Raffinose | Riboflavin |
| 126- | Rhamnose | Ribothymidine |
| 127- | Riboflavin | Scopolamine |
| 128- | Saccharopine | Sphinganine |
| 129- | Scopolamine | Sphingosine |
| 130- | Sepiapterin | Succinic acid |
| 131- | Spermine | Succinylacetone |
| 132- | Sphinganine | Taurine |
| 133- | Succinic acid | Threonic acid |
| 134- | Succinylacetone | Thymidine |
| 135- | Tartaric acid | Tiglylglycine |
| 136- | Terephthalic acid | Traumatic acid |
| 137- | Traumatic acid | Tricosanoic acid |
| 138- | Trimethylamine | Trimethylamine |
| 139- | Uracil | Umbelliferone |
| 140- | Urea | Uracil |
| 141- | Ureidosuccinic acid | Urea |
| 142- | Uric acid | Uric acid |
| 143- | Uridine | Uridine |
| 144- | Urocanic acid | Valproic acid |
| 145- | Valproic acid |  |
| 146- | Xanthosine |  |
| 147- | Xanthurenic acid |  |
